# Independent and sensory human mitochondrial functions reflecting symbiotic evolution

**DOI:** 10.3389/fcimb.2023.1130197

**Published:** 2023-06-14

**Authors:** George B. Stefano, Pascal Büttiker, Simon Weissenberger, Tobias Esch, Martin Anders, Jiri Raboch, Richard M. Kream, Radek Ptacek

**Affiliations:** ^1^ Department of Psychiatry, First Faculty of Medicine, Charles University and General University Hospital in Prague, Prague, Czechia; ^2^ Department of Psychology, University of New York in Prague, Prague, Czechia; ^3^ Institute for Integrative Health Care and Health Promotion, School of Medicine, Witten/Herdecke University, Witten, Germany

**Keywords:** mitochondria, virus, independent mitochondria, sentinel mitochondria, sensory mitochondria, exosomes, tunneling nanotubes, SARS-CoV-2

## Abstract

The bacterial origin of mitochondria has been a widely accepted as an event that occurred about 1.45 billion years ago and endowed cells with internal energy producing organelle. Thus, mitochondria have traditionally been viewed as subcellular organelle as any other – fully functionally dependent on the cell it is a part of. However, recent studies have given us evidence that mitochondria are more functionally independent than other organelles, as they can function outside the cells, engage in complex “social” interactions, and communicate with each other as well as other cellular components, bacteria and viruses. Furthermore, mitochondria move, assemble and organize upon sensing different environmental cues, using a process akin to bacterial quorum sensing. Therefore, taking all these lines of evidence into account we hypothesize that mitochondria need to be viewed and studied from a perspective of a more functionally independent entity. This view of mitochondria may lead to new insights into their biological function, and inform new strategies for treatment of disease associated with mitochondrial dysfunction.

## 1 Introduction: a blueprint for communication

Cells represent the fundamental units of life that have evolved to assemble into organisms of increasing anatomical, physiological and behavioral complexity. Each individual cell in our bodies is able to execute complex biological activities as an individual unit, as well as receive and process environmental cues, allowing it to function as a part of a tissue, an organ and ultimately an organism. To achieve this, cells need fuel and each eukaryotic cell incorporates energy-generating organelles called mitochondria. The mitochondria produce all the adenosine triphosphate (ATP), the basic biochemical carrier of energy needed to support eukaryotic life, earning them the label “the powerhouse of the cell”.

According to the prevailing hypothesis, mitochondria are of bacterial origin (Gray et al., 1999; Cavalier-Smith, 2006; Martijn et al., 2018; Munoz-Gomez et al., 2022). The endosymbiotic hypothesis proposes that mitochondria originated from specialized bacteria (i.e., α-proteobacteria) that formed a symbiotic interaction between mitochondria and their eukaryotic host cells, potentially by surviving endocytosis (Gray et al., 2001). Several lines of evidence support this hypothesis, as mitochondria are self-replicating, have circular DNA, and an independent transcriptional and translational machinery that resembles the one in modern day bacteria. The sequencing of various mitochondrial genomes, such as of the protist *Reclinomonas Americana*, has demonstrated that essential functions of electron-coupled ATP production and translation of mitochondrial proteins can be explicitly retraced to the bacterial ancestor (Gray et al., 2001). Both, bacterial DNA and mtDNA are compact in nature and contain unmethylated cytosine–guanine dinucleotide (CpG) motifs (Boguszewska et al., 2020). Furthermore, mitochondria and bacteria create and employ polycistronic transcripts, have clustered RNA genes, and notably, the mitochondrial transcription factor B2 (TFB2M) is homologous to the rRNA methyltransferase family in bacteria (Boguszewska et al., 2020). Importantly, similar to bacteria and viruses free metabolically competent mitochondria have been observed circulating in blood (Markova, 2017; Song et al., 2020). From an evolutionary perspective, eukaryotic cells that formed this symbiotic relationship gained a fitness advantage as mitochondria provided internal and continuous access to energy (ATP), ultimately resulting in complex behavior and cognition with high energy requirements (Raichle and Gusnard, 2002; Rangaraju et al., 2014; Magistretti and Allaman, 2015; Bruckmaier et al., 2020).

**Figure 1:**
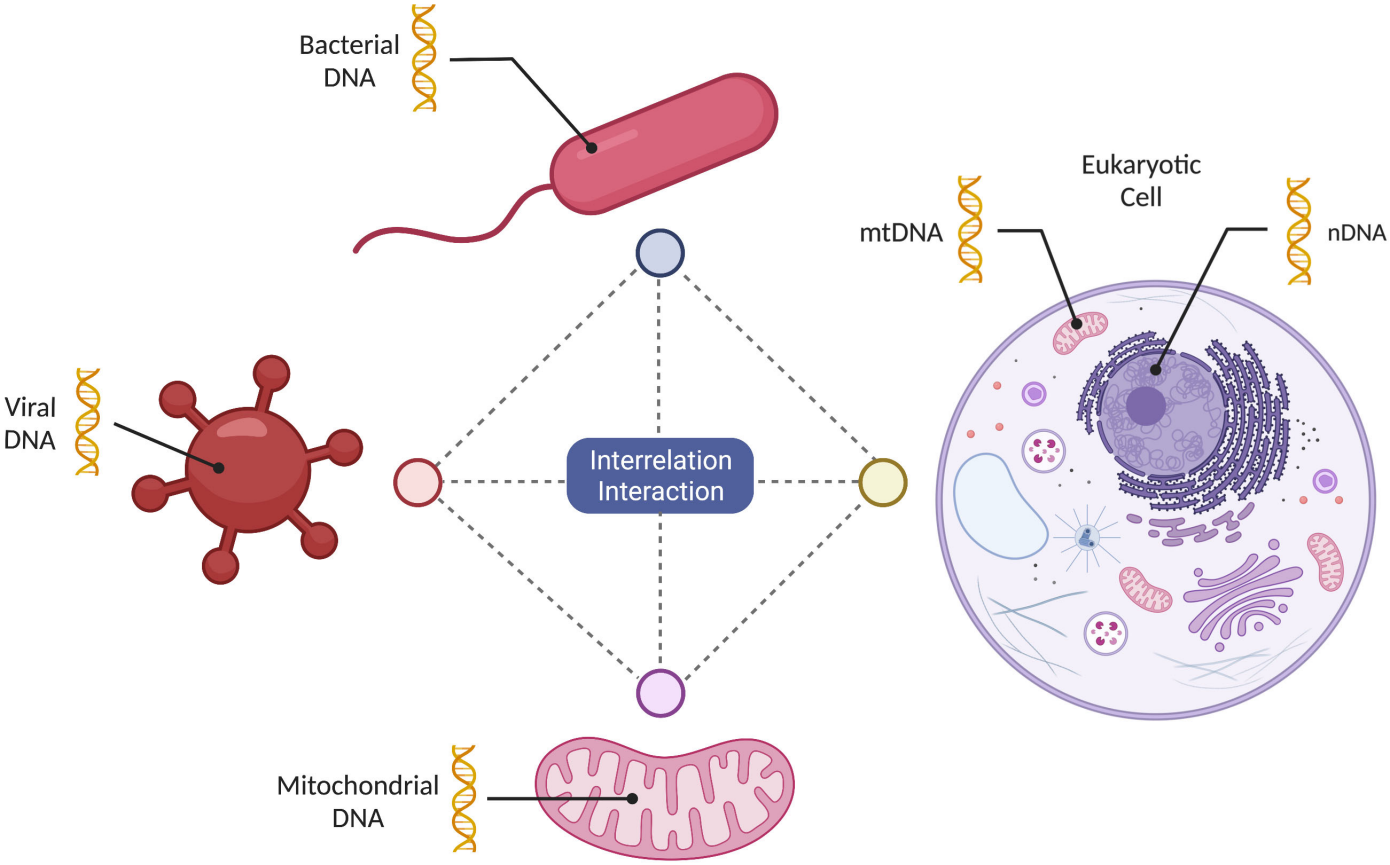
Mitochondrial multidirectional informational sharing. Mitochondria can share information in a multidirectional manner among themselves, as well as with bacteria, viruses and a eukaryotic host cell. This exchange of information is facilitated by the use of the “shared biochemical language” evolving simultaneously.

Mechanistically, the symbiotic relationship between eukaryotic cells and mitochondria is enabled by the universal biochemical “language”, a set of biochemical processes using a set of the endpoint molecules that remain universal across different cell types and diverse organisms (Stefano and Kream, 2022b) (**Figure 1**). These molecules facilitate multidirectional lines of communication inside the cell between mitochondria and the other cellular components, as well as among the cells in a given organism, and the host cells and microbes (i.e. bacteria and viruses) (Lobet et al., 2015; Han et al., 2019; Stefano and Kream, 2022b). This last type of interface between the host and the microbe is very often the one that is exploited by the pathogens that use common biochemical messengers to “hijack” cellular processes for their own fitness advantage, thus causing disease (Stefano et al., 2020). This interface includes host cell mitochondria, as pathogens can interact with and affect mitochondrial function (Wang et al., 2020; Stefano and Kream, 2022b). For example, SARS-CoV-2 hijacking of the mitochondria effectively diminishes innate and adaptive immunity by disrupting energy metabolism. Collected data indicate that activation of multiple Toll-like receptor (TLR) signaling pathways represents a unified mechanism that functionally integrates mitochondrial, bacterial and viral communication processes. For example, activation of TLR9 signaling pathways by extracellular mtDNA can promote downstream activation of p38 and mitogen activated protein kinase (MAPK) whose canonical substrates are unmethylated cytosine-phosphate-guanine (CpG) dinucleotides common to both bacteria and DNA viruses (Sorouri et al., 2022). Furthermore, the release of mitochondrial effectors mediated by B-cell lymphoma-2 family proteins may be linked to pyroptosis, a type of lytic cell death, which is a distinct response to bacterial and viral infections (Sorouri et al., 2022). Importantly, also shared mechanisms of action of clinically employed drugs against bacterial and viral infections may reflect complementary shapes or recognition domains indicating a shared biochemical language that has evolved simultaneously in different organisms. Given their mechanistic commonalities, it is no surprise that viruses, bacteria, and mitochondria can interact and effect changes that ensure their mutual survival in their microenvironment, demonstrating the power of the strategy. It is of interest to note that DNA transfer from mitochondria to the eukaryotic cell genome represents an old evolutionary phenomenon, preceding human speciation (Hazkani-Covo et al., 2010; Puertas and Gonzalez-Sanchez, 2020; Wei et al., 2022). However, recent research by Wei and colleagues demonstrates, there is an ongoing transfer of mitochondrial DNA into the nuclear containing genome (nuclear-mitochondrial segments (NUMTs) (Wei et al., 2022). Furthermore, methylation processes inhibited the expression of this genetic material, however, some segments, a minority, are expressed. We surmise this common phenomenon maybe involved in viral targeting of mitochondria, leading to eukaryotic cell genome targeting and access whereby aberrant proteins emerge. Here, this phenomenon may become more evident behaviorally in neurons coupled to cognition since they are susceptible to a diminished energy supply. However, unlike bacteria that are free living, mitochondria are symbiotic with the host cell and assumed to have no independent function.

Here, we will focus on discussing how the shared common lines of communication are used by the mitochondria to exert functional independence despite 1.45 billion years of endosymbiotic relationship. We will discuss how mitochondrial independence extends the symbiotic relationship, endowing eukaryotic cells, tissues and the organism with capability to evolve higher degrees of complexity. Furthermore, we will highlight examples of mitochondrial malfunction and how that contributes to pathology of many human diseases. We will build an argument that the current prevailing view of mitochondria as energy-generating organelles needs to be revised and expanded to recognize mitochondrial independence and sensory function. Thus, we provide an overview of the literature to support re-classifying mitochondria as a true dynamic symbiont with independent mobility, genetics, signaling/communication and ex-cellular functions.

## 2 Mitochondrial independence: the extension of the symbiotic relationship

### 2.1 Heteroplasmy and existence of extracellular mitochondria as factors supporting the mitochondrial functional independence hypothesis

A typical eukaryotic cell contains numerous mitochondria. For example, 30−40% of the cardiac muscle cell volume is occupied by mitochondria (Stefano et al., 2017). Each mitochondrion contains autonomous mitochondrial DNA (mtDNA), and transcriptional and translational machineries. Importantly, mtDNA mutational rates are 100-1,000 times higher than nuclear DNA (Wallace and Chalkia, 2013) therefore, given the number of mtDNA sequences that may exist within a cell, referred to as heteroplasmy, mtDNA harbors an enormous pool of differing genetic information that is closely tied to the survival of an organism (Collins et al., 2002; Tielens et al., 2002; Stefano and Kream, 2022a). Although mtDNA appears to be less complex than nuclear DNA, it functions in a similar way and can initiate metabolite-steered changes in gene expression between bacteria, mitochondria, and the host cell’s nucleus (Han et al., 2019). Importantly, by preserving a separate genome, mitochondria have maintained a degree of autonomy, as we discuss throughout this article.

Historical studies have described functional and morphological heterogeneity of populations of mitochondria sorted according to cell type and differential aerobic/anaerobic conditions (Collins et al., 2002; Tielens et al., 2002). These collected data support the existence of distinct populations of cellular mitochondria that also function anaerobically to produce ATP with coupling of electron transport in low oxygen conditions (Takahashi and Sato, 2014; MacVicar et al., 2019). This form of individualized communication depends on factors such as the location of the mitochondria within a cell, the host cell type, and their microenvironment. This diversity of mitochondrial genomes also suggests that mitochondria may be able to function independently outside their host cell, and recent research has provided evidence for the presence of functional mitochondria in the extracellular environment (Pollara et al., 2018; Al Amir Dache et al., 2020; Song et al., 2020). The existence of free and exosomal metabolically competent mitochondria, mitochondrial proteins, and proteolipid fragments, has been demonstrated in human and murine cerebrospinal fluid (CSF) after injuries (i.e., ischemic stroke or hemorrhage) (Hayakawa et al., 2016; Chou et al., 2017). Importantly, Joshi and coworkers demonstrated stimulated release of functional, dysfunctional, and fragmented mitochondria into the extracellular neuronal milieu, thereby highlighting the biological importance of their specific ratios under pathological conditions (Joshi et al., 2019). These observed phenomena support the hypothesis that mitochondria can survive and function independently of their cellular host (Chandel, 2014; Snyder et al., 2015; Bohovych and Khalimonchuk, 2016; Angajala et al., 2018; Lynch, 2020). We speculate that extracellular mitochondria may represent a healthy reservoir of the integration-ready symbionts that can enter compromised cells and replace damaged mitochondria. If true, this would have major implications for our understanding of basic cell biology as well as a number of diseases, such as diabetes, neurological issues, psychological, epilepsy, and cancer, which have been associated with mitochondrial dysfunction (Stefano and Kream, 2015a; Hayakawa et al., 2016; Main and Minter, 2017; Schilf et al., 2021).

Intact and fragmented mtDNA and additional chemical components of mitochondria have been previously observed to promote innate immune responses via selective activation of cellular signaling such as the anti-viral cGAS–STING–TBK1 pathway (Wu et al., 2019). Interestingly, cellular release of mtDNA and associated damage products has been proposed to engender a beneficial selective signaling response that promotes nDNA repair in affected cells, thereby suggesting that mtDNA represents an important sentinel of genotoxic stress (Wu et al., 2019). This helps the cell eliminate the chemical stressors that could damage the cell’s DNA or internal structures. In this sense, mitochondria act as “sentinels” in an intracellular immune system, providing an early warning signal for the detection of intracellular perturbations that affect the cellular energy supply (Chandel, 2014; Stefano and Kream, 2015b; Bohovych and Khalimonchuk, 2016; Saint-Georges-Chaumet and Edeas, 2016; Dutta et al., 2020; Tiku et al., 2020; Brokatzky and Hacker, 2022). Furthermore, if this distress signal is of sufficient strength, the scope of the immune response increases to the point where adjacent cells may be alerted to the danger. The distress signal in this protective feedback loop is likely initiated by mitochondria-derived reactive oxygen species (ROS), such as hydrogen peroxide. Therefore, mitochondria can perform both positive and negative sensory functions, by not only detecting a perturbation but then channeling this determination into an established reaction.

Although mitochondrial function is tightly linked to oxygen levels and believed to depend on its presence, they can also continue to function and generate energy under low oxygen (hypoxic) conditions (Kalghatgi et al., 2013; Stefano et al., 2015c; Debattisti et al., 2017; Castillo et al., 2019; Lynch, 2020). Mitochondria have the ability to “shift” from aerobic to anaerobic functional capacities in invertebrates and mammalian tissues (Stefano et al., 2015c; Záhonová et al., 2022). This potentially demonstrates an aspect of independent behavior in the response to microenvironmental challenges and their nature as a dynamic cellular entity ensuring survival by exhibiting a higher level of performance to meet stressful stimuli. Mitochondria are known to transform into anaerobic mitochondria, hydrogenosomes, mitosomes, and various transition stages in between, collectively called mitochondrion-related organelles (MROs), which vary in enzymatic capacity (Záhonová et al., 2022). Protists that experience hypoxia often possess metabolically distinct MROs. Over the past decade, deep-sequencing studies of free-living anaerobic protists have revealed novel configurations of metabolic pathways that have been co-opted for life in low oxygen environments (Stairs et al., 2015). Utilization of these anaerobic pathways of mitochondrial energy metabolism known to be present in other mammalian tissues may provide strategies to limit mitochondrial dysfunction and allow cellular repair before the onset of irreversible injury, for example, by ischemia or hypoxia (Weinberg et al., 2000). Similar evolutionary conserved protective mechanisms can be found in mammalian oocytes, where maternally inherited mitochondria remain functionally silent (i.e., quiescent state) in order to minimize mitochondrial DNA mutations and their passing down to the embryo (Babayev and Seli, 2015). Quiescent mechanisms are believed to rely on various highly complex metabolic shifts depending on cellular-mitochondrial communication and cell-cycle status and involve the activity of specific metabolic proteins (e.g., mitochondrial fusion protein optic atrophy 1 [OPA1]), mitochondrial fragmentation, the regulation of mitochondrial ROS and antioxidant activity, etc. (Coller, 2019; Baker et al., 2022). Interestingly, paternal (i.e., sperm) mitochondria may exert similar protective functions by sacrificing themselves early in the fertilization process (i.e., gamete fusion) in order to protect the embryo from deleterious ROS-induced mutations, which they may carry (Babayev and Seli, 2015).

As our work suggests, a hypoxic microenvironment may also be the ultimate trigger of the intracellular mitochondrial response (Stefano and Kream, 2015b; Esch et al., 2020), and that under those conditions the cells recruit new mitochondria from the extracellular pool. We surmise these recruited mitochondria may in turn serve an early surveillance function (forming part of a protective feedback loop) and have a role in chronic inflammation (Esch et al., 2020; Stefano et al., 2022c). Furthermore, recent work has demonstrated that it is possible to transplant healthy mitochondria and rescue the function of damaged cells, tissues, organs, and even organisms (McCully et al., 2016; Hayakawa et al., 2018; Nakamura et al., 2020). Together with growing ability to edit the mitochondrial genome using CRISPR-based genome editing approaches (Wallace, 2020), this suggests that some of the diseases caused by mitochondrial dysfunction may be repaired and treated by replacing malfunctioning mitochondria with engineered ones. Overall, the discovery of extracellular mitochondrial pool as well as the genetic independence led us to propose that unlike other organelles that can’t survive outside the cell, mitochondria may have a cell-independent extracellular function.

### 2.2 Mitochondrial mobility as a factor supporting the mitochondrial functional independence hypothesis

**Figure 2:**
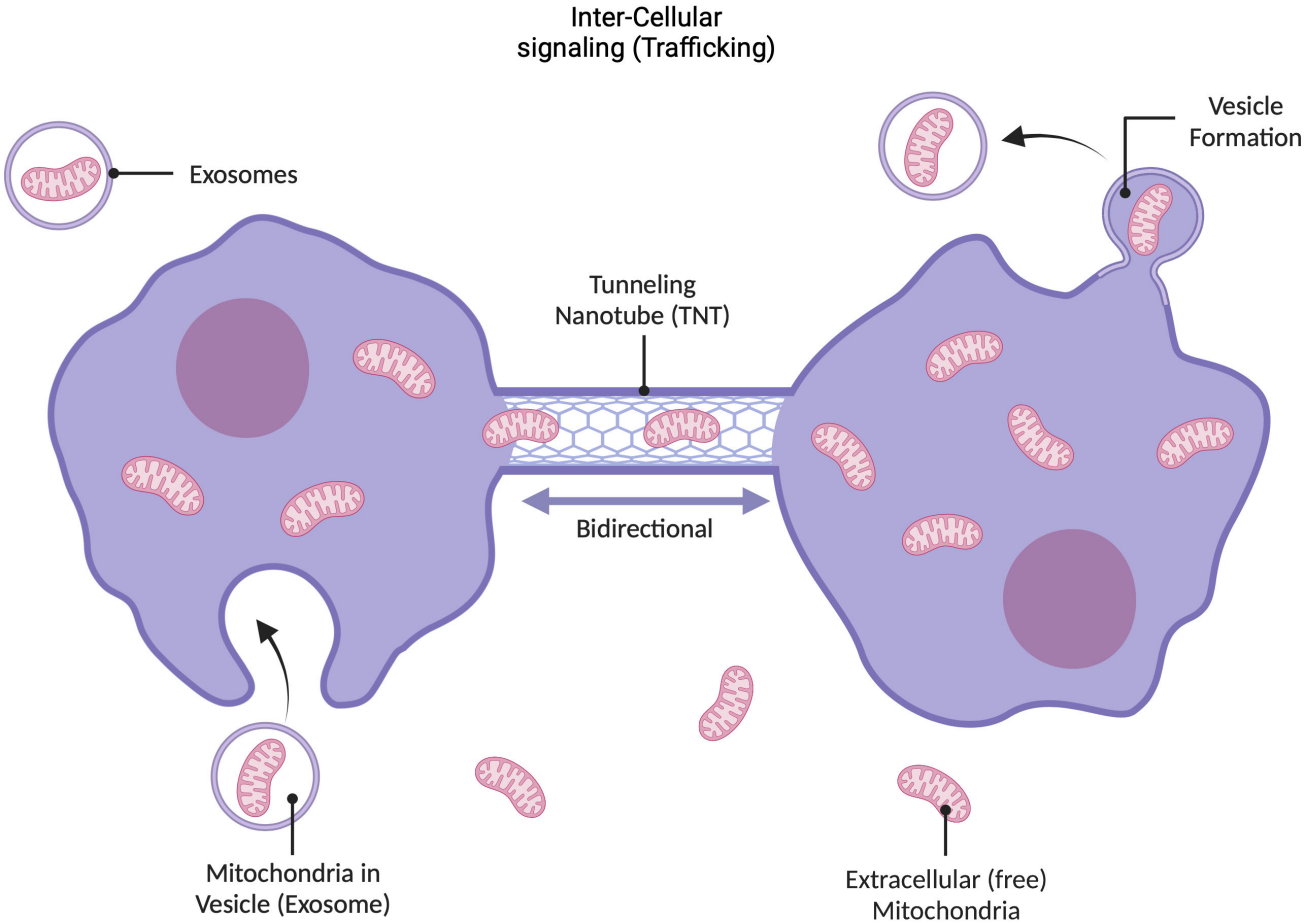
Dynamic nature of mitochondria. Mitochondria are dynamic entities that exhibit multiple kinds of motion in response to external cues and intracellular environment. For example, if a region of the cell becomes disrupted, healthy mitochondria can migrate to that area. Mitochondria also travel between cells, using exo- and endo-cytosis pathways, and extracellular mitochondria have independence to move through extracellular space, for example, to reach compromised areas in the case of cerebral ischemia, hypoxia, etc. (Hayakawa et al., 2016). There are two primary mechanisms through which mitochondria can traffic between cells: they can enter into membrane-enclosed vesicles called exosomes that are secreted by the cell, which also contain mtDNA; or they can travel through cellular tunneling nanotubes. Recently it has also been suggested that mitochondria and their products can make use of gap junction channels for intercellular transfer. This mobility demonstrates that mitochondria are not bound to the cell in which they were formed, and may serve as mobile emergency battery (energy) and sensory units (see text).

Mitochondria can move around in their intracellular environment in response to cellular stressors such as hypoxia (Berridge and Neuzil, 2017; Debattisti et al., 2017; Wu et al., 2019; Gitschlag et al., 2020). Recent studies have demonstrated that mitochondria can also move from one cell to another to perform the same type of restorative function (Birsa et al., 2013) (**Figure 2**). One mechanism by which mitochondria can leave and enter a cell involves the mitochondrion being encapsulated in the cell membrane and removed from the cell via exocytosis in membrane-bound structures called exosomes (McCoy-Simandle et al., 2016). Another mechanism involves the trafficking of mitochondria between cells via tunneling nanotubes (Birsa et al., 2013; Hayakawa et al., 2016; Jackson et al., 2016). Recently, it has been suggested that intercellular transfer of mitochondria and their products may also occur via gap junction channels (Liu et al., 2022). Additionally, in a damaged cell, newly recruited mitochondria may supply genetic information to existing mitochondria or take their place in the execution of energy- and repair-associated processes (Stefano and Kream, 2022a; Stefano and Kream, 2022b). For example, observations of intercellular migration of mitochondria from neurons to astrocytes and from astrocytes to damaged neurons and the transfer of endothelial progenitor cell (EPC)-produced extracellular functional mitochondria to brain endothelial cells support these contentions (Nakamura et al., 2020). Thus, mitochondria may serve as “ambulances” by sensing and responding to microenvironmental emergencies that could lead to complications at the tissue or organ level. This behavior also suggests that mitochondria have retained their independence to leave a given cell or environment and appear where they are most needed, sensing the “traumatized area”. Collectively, these studies suggest much more complex mobility capabilities than reported for other organelles, further supporting our view of mitochondria as independently functioning units.

### 2.3 Mitochondrial communication as a factor supporting the mitochondrial functional independence hypothesis

The recent insight that mitochondria engage in multiple types of intracellular and intercellular communication led to a new label of mitochondria as a “social” organelle (Picard and Sandi, 2021). Some of the features of mitochondrial behavior that have been described are coordinated intracellular movement, either driven by cellular cues or mitochondria themselves, mitochondrial fusion and fission, group formation and synchronized behavior (Picard and Sandi, 2021). All these processes depend on multiple different lines of communication that mitochondria seem to employ, such as direct interaction between mitochondria, or between mitochondria and other organelles, whereby membrane contacts enable direct transmission of information (Raimundo and Krisko, 2019; Xia et al., 2019; Desai et al., 2020). Furthermore, mitochondrial mobility from one cell to another described above opens further communication pathways (Wang and Gerdes, 2015; Jackson et al., 2016; McCoy-Simandle et al., 2016). Very recently mitochondria-plasma membrane (PM) interactions have been discussed as critical, yet understudied, mediators of mitochondrial independence, suggesting that mitochondria should be viewed as more than organelles subsumed by the cell (Montes de Oca Balderas, 2021). That review also issued a call to action for more study of mitochondria-PM interactions and their biological roles.

Mitochondria also engage in active inter-mitochondrial communication to coordinate self-assembly and dynamics, which mirrors some aspects of quorum sensing of bacteria (Picard et al., 2015). In this case, communication is enabled by formation of inter-mitochondrial junctions that facilitate electrochemical communication. All these levels of communication, inter-mitochondrial, inter-organelle, and inter-cellular are reminiscent of different types of communication that a cell engages in. Thus, mitochondrial ability to maintain a “social” network and engage in different types of communication, supports our hypothesis that mitochondrial behavior and functions are significantly more independent than currently viewed, given that mitochondria are capable of interpreting signals and making autonomous decisions, as well as collective (quorum-like) decisions. It has been previously demonstrated that this social behavior extends across kingdoms, facilitating communication with other symbionts, such as bacteria and viruses. For example, recent findings demonstrate that chemical signals from bacteria (i.e., colonic acid and methyl metabolites) can regulate mitochondrial fusion and fission thereby modulating gene expression in the nucleus (Han et al., 2019).

Collectively, the main factors we highlight here, heteroplasmy, presence of functional extracellular mitochondria, mitochondrial mobility and mitochondrial “social” network and communication support our hypothesis that mitochondria exhibit a high degree of functional independence from their host cells. This strongly suggests that mitochondria are more than just organelles and, as proposed, represent independent (or semi-independent) functional entities. Although evidence for mitochondrial independence is robust, further studies are needed to better understand mitochondria from this new perspective, especially the role of free metabolically competent mitochondria in human physiology and disease.

## 3 Implications of mitochondrial functional independence hypothesis for understanding and treating disease

Numerous diseases affect mitochondrial function or arise due to mitochondrial abnormalities. Indeed, mitochondrial dysfunction is currently associated with over 300 different conditions, including cardiac disease and diabetes (Arnoult et al., 2003; Stefano and Kream, 2015a; Stefano et al., 2017; Pollara et al., 2018; Lynch, 2020; Tiku et al., 2020; Schilf et al., 2021). Thus, studies of how mitochondria function in different environments and under varying conditions have the potential to reveal new treatment strategies for diseases like cancer, heart disease, and mental illness. From a perspective of our working hypothesis, it will be of interest to examine what type of autonomous mitochondrial activity contributes to disease, as well as provide evidence that extracellular mitochondrial pool serves as a reservoir of ready-made mitochondria that can rapidly replace malfunctioning ones in affected cells.

As discussed above, mitochondria engage in multiple interactive regulatory signaling pathways across cytosolic and nuclear cellular compartments and with commensal and pathogenic bacteria in the well-studied gut microbiome and in the blood (Lobet et al., 2015; Snyder et al., 2015; Bedarf et al., 2017; Markova, 2017; Castillo et al., 2019; Han et al., 2019; Al Amir Dache et al., 2020; Deo et al., 2020; Esch et al., 2020; Buttiker et al., 2021a; Buttiker et al., 2021b). This bacteria-mitochondria exchange is especially likely to affect the extracellular mitochondrial pool. Possible outcomes of these interactions are incorporation of rogue genetic material or physical colonization that negatively influence mitochondrial behavior in a way that benefits the pathogen. Horizontal gene transfer (HGT) across organisms, for example, for self-preservative purposes is a well-documented phenomenon. For instance, HGT with chromosomal transfer of the *E. faecalis* pathogenicity island containing protective genes is an example of inter-bacterial gene transfer and accounts for the high resistance of *Enterococcus faecalis* (Emamalipour et al., 2020). Furthermore, sequence analysis of tardigrades, microscopic eukaryotic animals, demonstrated that a substantial fraction of their genes (i.e., ca. 17.5%) have been obtained from bacteria, plants, fungi, and archaea, confirming HGT also between kingdoms. Another example of eukaryote-bacteria HGT is the Gram-negative bacteria *Agrobacterium tumefaciens* which transfers and merges DNA into plant genomes causing crown-gall disease. Inter-eukaryotic HGT of mitochondrial genes and encoding ribosomal and respiratory proteins has been demonstrated in a phylogenetic analysis of different plant species (Bergthorsson et al., 2003). While HGT has been demonstrated to occur in many biological systems, gene transfer between bacteria and mitochondria in mammals has not extensively been examined. Milner and coworkers identified a functional type II restriction modification system in the mitochondrial genome of a marine protist acquired via HGT from bacteria (Milner et al., 2021). Furthermore, Rice and Palmer performed next-generation sequencing to putatively demonstrate HGT in plastids of related genes (e.g., rpl36) which are found in cyanobacteria and other groups of bacteria (Rice and Palmer, 2006). Accordingly, Rice and coworkers have suggested that donor mechanisms between bacteria and plastids may underlie the targeting of plant mitochondria and the transfer of regional and entire mtDNA genomes, as reported in the plant *Amborella trichopoda* (Rice et al., 2013).

Viruses have also been shown to affect mitochondria in a similar way (Saint-Georges-Chaumet and Edeas, 2016; Bedarf et al., 2017; Han et al., 2019; Neu and Mainou, 2020; Singh et al., 2020; Stefano et al., 2022c); Stefano and Kream, 2022a; Stefano and Kream, 2022b. Integration of viral genomes into the human genome has been demonstrated for the human immunodeficiency virus type 1 (HIV-1) and ancient human endogenous retroviruses (HERVs), which make up 8% of the nuclear genome (Diaz-Carballo et al., 2017). These molecular events are mediated by actions of reverse transcriptase that can also lead to drug resistance (Megens et al., 2014). Double stranded RNA has also been detected in human mitochondria following SARS-CoV-2 and other coronavirus infections, confirming the exchange of information via outer mitochondrial membrane protein interactions (e.g., Tom20) (Mehrzadi et al., 2021; Shang et al., 2021). In sum, collected data suggest that HGT from bacteria to mitochondria is a rare occurrence. Furthermore, it appears that HGT from viruses to mitochondria has not been previously described in the biomedical literature.

Given the quantity and diversity of genetic information found in bacteria, viruses, and mitochondria, we envision that a constant multidirectional exchange of information occurs between these entities (**Figure 1**). However, the potential roles of this dynamic and complex process, beyond providing survival advantage for the pathogen, remain to be determined. For example, interesting questions related to microbiome-dependent increase in mitochondrial genetic diversity are whether these changes compromise mitochondrial energy supply function and fitness of the host, and whether they are retained and transmitted through multiple generations of mitochondria and the host.

These insights raise the possibility of subverting this multidirectional communication between the mitochondria and the gut microbiota, including the virome, to engineer features that can be incorporated into healthy extracellular mitochondria as a strategy to thwart disease. On the flip side, it appears that macrophages, an evolutionarily ancient subset of immune cells, regularly release their mitochondria into the extracellular space to facilitate the distribution of energy sources in the bacteria-rich proinflammatory gut environment, which is then “hijacked” by bacteria to exasperate infection (Saint-Georges-Chaumet and Edeas, 2016; Main and Minter, 2017; Buttiker et al., 2021b). We speculate that after exchanging information with bacteria, the extracellular mitochondria may re-enter macrophages, and get transported elsewhere in the body via the circulatory system (Stefano et al., 1994). If these macrophages, carrying mitochondria loaded with bacterial DNA, were to enter the vascular elements of the brain, they may induce a proinflammatory response, which negatively affects normal mental capacity by reducing the oxygen supply to neuronal cells. In this example, multidirectional communication between bacteria and mitochondria functions not only as an immunological “sentinel” but also as a reservoir of information that bacteria can access to influence mitochondrial function. Additionally, cell trafficking into the brain suggests that the blood brain barrier can be used as an access point into the central nervous system (Stefano et al., 2022d). Bacterial-mitochondrial communication could also result in mitochondrial heteroplasmy, as previously touched upon, whereby individual mitochondria serve as a reservoir of genetic information and this genetic diversity enables the mitochondria to meet multiple challenges. In this way, heteroplasmy may be an evolutionary strategy for promoting health and longevity (Stefano and Kream, 2022a; Stefano and Kream, 2022b).

Mitochondrial heteroplasmy has been shown to exert profound effects on downstream mitochondrial protein expression via alterations on transcriptional regulation of mitochondrial gene expression (Ye et al., 2022), and selective mutations in mitochondrial tRNA genes (Abbott et al., 2014). Interestingly, it has been empirically demonstrated that the mitochondrial protein ATFS-1 promotes the binding of the mtDNA replicative polymerase (POLG) to mtDNAs many of which express heteroplasmic somatic mutations (Yang et al., 2022). Furthermore, these data indicate that ATFS-1 promotes mtDNA replication in dysfunctional mitochondria via enhanced POLG-mtDNA binding, which will certainly exert downstream effects on restoration of mitochondrial protein expression.

Functional independence of mitochondria has important additional implications for treatment of human disease. For example, mitochondrial transplantation has been explored as a strategy to provide a remedy for replenishing dysfunctional mitochondria (Valenti et al., 2021). Mitochondrial transplantation might be useful in a number of disorders, such neurodegenerative diseases, toxic injury, ischemia, cardiovascular diseases, aging, type 2 diabetes, and cancer (Park et al., 2021; Ulger and Kubat, 2022). In regard to ischemia treatment, a systematic review of the animal and human trials for mitochondrial transplantation found beneficial effects of mitochondrial transplantation. Various ways of mitochondrial transplantation have been tested, most of which involve injection of isolated autologous, allogeneic, or even xenogeneic mitochondria or their particles directly into the affected area or into the bloodstream (i.e., intravascularly) (Nakamura et al., 2020; Liu et al., 2022). The therapeutic potential of mitochondrial transplantation has been signified in various murine models. Specifically, the injection of healthy mitochondria demonstrated improvement of acute bioenergetics in spinal cord injury, the prevention of cognitive impairment in Schizophrenia, the attenuation of dopaminergic neuron degeneration in Parkinson’s Disease, and induction of neuroprotective effects following cerebral ischemia (Nakamura et al., 2020; Liu et al., 2022). However, clinical translation of mitochondrial transplants remains limited, and further research is required to demonstrate efficacy and safety of these procedures (UMIN000043347) (Hayashida et al., 2021). Therefore, mitochondrial transplantation is currently under debate and many questions remain open (Lightowlers et al., 2020). Nonetheless, the fact that mitochondria can be transplanted, and result in cells with repaired function further supports our hypothesis that mitochondria are functionally independent.

Taken together, the communication between mitochondria, bacteria and viruses may drive ongoing evolution of mitochondria. In some cases, integrating bacterial or viral genetic information into mitochondrial population may drive infection and benefit the pathogen. However, we also argue that some of these changes may increase the fitness of the host as well. Lastly, we envision mitochondrial engineering as a promising biotechnological strategy, as well as a potential therapeutic modality for treatment of human disease, and promotion of health and longevity. We appreciate that at this point this remains a speculation, and that applications aimed at extending lifespan are controversial and deserve careful ethical consideration. Nevertheless, we expect that the new view of mitochondria as independent entities will open future opportunities for such advancements to widen our understanding of basic biology and potentially translate this insight into new medicines.

A 2016 publication by Wang and coworkers indicated that there was a paucity of approved drugs that directly targeted mitochondria for therapeutic intervention in a wide variety of human disorders, notably metastatic disease and metabolic insufficiencies (Wang et al., 2016). Recent extensive reviews, however, present positive developments and linked to multiple medicinal chemical candidates for treatment of a broad spectrum of cancer cells and solid tumors (Singh et al., 2021) and multiple metabolic disorders (Catalan et al., 2020) via direct targeting of mitochondria. For example, Singh and coworkers have focused on mitochondrial inhibitors of dysregulated bioenergetics processes in cancer stem cells to inhibit progression of a wide variety of human cancers (Singh et al., 2021). Potential agents include novel transport vehicles in combination with known pharmacophores, including natural products as well as experimental compounds and prodrugs. The authors provide strong commentary on the unique biochemical properties of cancer stem cell mitochondria which have previously made them quite refractory to traditional treatments and should now be approached via novel choice and testing of potential matching of therapeutic agents. Catalan and coworkers highlight recent pharmacological advances in the treatment of human pathologies linked to mitochondrial dysfunction arising from genetic polymorphisms affecting multiple phases of mitochondrial protein and nucleic acid expression or dysregulation of convergent metabolic pathways negatively affecting mitochondrial bioenergetics (Catalan et al., 2020). They provide a useful compendium of both potential targeting and non-targeting mitochondrial agents. In conclusion, it is clear that the interactive complexity of mitochondria that is required to support normative energy metabolism requires development of novel agents and/or the use of old agents that did not initially target them, e.g., antibiotics and natural compounds for inhibition of cancer progression and treatment of genetically determined and systemic metabolic disorders.

Furthermore, the mitochondrion itself may be considered a highly specialized nanoparticle approaching an upper limit of 1000-2000 nanometers in diameter. From mechanistic perspectives it may contain hundreds of restorative circular mtDNA molecules, mRNAs encoding restorative mitochondrial proteins and unique tRNAs (Xu et al., 2022). Accordingly, emerging restorative technologies involving mitochondrial transplantation should be intimately linked to the strict maintenance of the integrity of essential biochemical and molecular components of this unique “nanoparticle”. From traditional pharmacological perspectives, however, cancer therapeutics utilizing nanoparticles have attempted to specifically target and destroy cancer cell mitochondria via photodynamic, photothermal, or direct chemical approaches (as reviewed, (Xu et al., 2022)). Here the authors contend that effective targeting and destruction of cancer stem cell by nanoparticle administration have been demonstrated to be highly efficacious compared to non-mitochondria-targeting platforms. Importantly, mitochondrial targeting by nanoparticles must be effectively designed to accommodate ongoing tumor microenvironments for highly efficacious cancer cell destruction and represents a promising strategy for drug delivery to inhibit tumor progression.

## 4 Conclusions

Although bacterial origin of mitochondria has been widely accepted, most current research and thinking in the field assumes that mitochondria have lost independent function and become fully integrated into eukaryotic cells; thus, mitochondria are rarely viewed as anything but an organelle that provides energy to support life. However, here we argue that the view of mitochondria needs to be expanded to consider mitochondria as an independent entity, especially given the recent discovery of fully functional extracellular mitochondria. We summarize the evidence that mitochondria have numerous independent functions, and may, to some extent, evolve independently of the host through interactions and exchange of genetic information with bacteria and viruses. In our current model of expanded, independent mitochondrial function we propose that extracellular mitochondria play a key role in human physiology as an on-demand reservoir of healthy mitochondria that can be rapidly internalized to replace damaged and faulty ones in response to a range of environmental signals. In conclusion, the well-established symbiotic relationship that exists between the mitochondria and the eukaryotic host cell has masked the other intriguing features of this system and its independent nature and sensory function, both of which merit further investigation.

Clearly, organismic independence is both a key scientific as well as a philosophical question that requires carefully designed sets of definitions. Many different qualitative propositions have been offered to complete or challenge the Darwinist view of defining a biological independent entity (Gould and Lloyd, 1999). Depending on the precise definition, the organismic independence of many procaryotes and other microorganisms may be functionally linked to their strict dependence on a symbiotic or parasitic relationship. Although we do not suggest that the mitochondrion may or may not be a biologically independent entity, similar philosophical conclusions could be drawn, given the presented mitochondrial mechanisms, the individual genomic information, as well as the organelle’s reproduction via prokaryotic binary fission. In this paper, however, we encourage the reader to challenge the current position of the mitochondrion and appreciate its functional independence within the constraint of its human host.

## Author contributions

Conceptualization, GS, RK; investigation, PB, SW, TE; writing—original draft preparation, GS; writing—review and editing, RK, SW, TE, MA, JR, RP; visualization, PB, GS; supervision, SW, RP; project administration, JR, MA; All authors have read and agreed to the published version of the manuscript.
